# A systematic review of actions aimed at university students with ADHD

**DOI:** 10.3389/fpsyg.2023.1216692

**Published:** 2023-07-07

**Authors:** María Álvarez-Godos, Camino Ferreira, María-José Vieira

**Affiliations:** Department of Psychology, Sociology and Philosophy, University of Leon, Leon, Spain

**Keywords:** attention deficit hyperactivity disorder, guidance, actions, higher education, university

## Abstract

University students with Attention Deficit Hyperactivity Disorder (ADHD) need to have a range of actions at their disposal that are tailored to their needs. The aim of this study is to analyse the actions of support for university students with ADHD by means of a systematic review of ERIC, WOS, and Scopus from 2017 to 2022, following the PICOC and PRISMA guidelines. A sample of 24 studies are analysed through the MAXQDA 2022 software. The results show two types of studies, on the one hand, supporting actions offered directly by universities to their students and, on the other, intervention programs from which university students have benefit but outside the university settings. Concerning the first type, universities mainly offer accommodations linked to exams, tutoring and online courses adapted to ADHD students. About the second type, programmes focused on cognitive-behavioural therapy, coaching and mindfulness have proven to be useful with ADHD university students. In this sense, further research is needed to analyze the viability of including these intervention programs for ADHD students at universities.

## 1. Introduction

Attention Deficit Hyperactivity Disorder (ADHD) is characterised by the appearance of inattention/hyperactivity-impulsivity constantly in the subject, affecting their development (American Psychiatric Association, 2013). The presence of hyperactive–impulsive symptoms is more usual in children and preadolescents, while the inattentive subtype is perceived to a greater extent in adolescence and adult life (Balbuena, 2016). ADHD is associated with poor academic performance across the lifespan, affecting individuals from childhood to adulthood. Symptoms of inattention and deficits in executive functioning play a key role in academic challenges, while hyperactivity/impulsivity and conduct problems are not strongly associated with academic difficulties (Daley and Birchwood, 2010). Additionally, ADHD has a broad comorbidity with a few other disorders. It can largely be found associated with oppositional defiant disorder, conduct disorder, learning disorder and disruptive mood dysregulation disorder. In a smaller number of cases, it is associated with antisocial personality disorder and intermittent explosive disorder, and unusually with tic disorder, obsessive–compulsive disorder, and autism spectrum disorder (American Psychiatric Association, 2013). According to a longitudinal study conducted by Sibley et al. (2017), rates of persistence of ADHD into adulthood vary substantially, depending on the procedure used to gather and analyse information, which includes structured interviews and rating scales along with the use of self-reported or parent/other-reported information as well as the selection of symptom threshold. With regards to the diagnosis of ADHD in young adults, structured interviews help to gather self-reports from the young adults when parental information is not available. Nevertheless, combining parental reports with self-reports obtained from assessment scales achieves an optimal balance between sensitivity and specificity. Estimates of the prevalence of this disorder in adulthood tend to range from 3–5% (Kessler et al., 2005; Polancyzk et al., 2007), but this is likely to vary depending on the methodology employed in the earliest stages of diagnosis. This has implications for more precise diagnosis in future educational stages, such as higher education.

University students diagnosed with ADHD revealed compelling evidence that the prevailing symptoms inherent in this neurodevelopmental condition exhibit a strong correlation with elevated levels of stress, thus emphasizing the significant impact that ADHD has on the psychological well-being and emotional equilibrium of affected individuals (Harrison et al., 2013). Such stress can also be associated with the different demands that the access to Higher Education entails for students with ADHD, since it implies that university students have sufficient levels of executive functioning and autonomy, as well as a good working memory and planning in their studies (Garello and Rinaudo, 2013), skills that people with ADHD do not have highly developed, affecting their academic performance. In addition, in this new stage of their lives, university students with ADHD face a double challenge, due, on the one hand, to the symptoms of the disorder itself and, on the other, to the presence of a new social context, the university, where they assume temptations and distractions, and where the supervision of their parents decreases in relation to the previous educational stages. They face several challenges that make it difficult for them to adapt, such as new autonomy, increased distractions, dealing with a new structure and schedule for their studies, and reduced supports (Fleming and McMahon, 2012; Knouse and Fleming, 2016; *LaCount et al., 2018).

In spite of this difficulties, the academic challenge at university level appears to pose significant barriers for individuals with ADHD, specifically with regard to executive functions, metacognition, and emotional regulation, all of which impact learning processes (Varrasi et al., 2022). High levels of ADHD symptomatology and low levels of executive functioning cause these students to suffer a negative relationship with their impairment, which in turn, leads to low self-esteem (Dorr and Armstrong, 2019). Even, in studies where no significant alterations were found on standardized tests of executive functioning in adults with ADHD, they themselves rated their executive abilities as impaired (Butzbach et al., 2021). To this end, reducing ADHD symptoms in university students allows improving their executive functions through intervention programs (Salvi et al., 2019). In sum, ADHD negatively affects academic performance and achievement, leading students with this disorder to drop out of university due to poor grades compared to their peers (DuPaul et al., 2018). Firstly, one of the factors that lead university students with ADHD to academic failure is the lack of specific support within the university environment, since the support they receive at higher education is less in relation to support offered in previous educational stages (Kwon et al., 2018).

As a general offer to all students, universities offer support through guidance services that cover three main areas (Watts and Van Esbroeck, 1998):

- Academic guidance: offering learning aids and educational support.
- Personal guidance: on personal, social, and emotional issues.
- Career guidance: focusing on the introduction to working life.

With regard to academic guidance, this support is developed within teaching by academic staff or in the form of central academic services. Academic services offer support in writing, reading, mathematics, taking tests, and time management, based on a series of educational actions (Gregg, 2009). On the other hand, personal guidance focuses on individual support to improve personal efficacy with an impact on self-esteem and self-awareness (Everitt et al., 2018). Finally, career support focuses on helping students to manage the choices and transitions they need to make as they exit their courses of study (Watts and Van Esbroeck, 2000).

This support offered to all university students regarding academic, personal and career concerns must be adjusted to students with disabilities, attending to their specific needs, as is the case of university students with ADHD. To meet the needs of students with disabilities, universities have created administrative offices where students look for support (Getzel, 2008). Most universities have a responsible person whose role is to take care of students with disabilities within the university, so a student with ADHD has the right to contact this person to know what support they may need, so that they can collaborate with his/her teachers to make them happen (Barkley, 2010).

Specifically, university students with ADHD often need a series of accommodations that are reasonable to their needs to promote their academic performance, improve their results, and to benefit from these accommodations to improve their executive functioning. For this reason, higher education institutions have been forced to offer them effectively to their students in need (Kettler, 2012; Budd et al., 2016).

These accommodations are defined as:

Changes to practices in schools that hold a student to the same standard as students without disabilities but provide more benefit to students with a disability (i.e.: differential boost) to mediate the impact of the disability on access to the general education curriculum (Harrison et al., 2013, p. 556)

Another type of support for students with ADHD is intervention programmes, usually carried out by psychologists or other professionals in collaboration with different specialised agents aimed at treatment in areas such as development of the personality, executive functions, emotional disorders, following specific protocols and control measures (Solé, 2005).

The main objective of this study is to describe the support actions aimed at university students with ADHD at the international level.

## 2. Methods

The PICOC principles were followed: Population, Intervention, Comparators, Outcomes, Context (Maier, 2013) to determine the research question and the characteristics of the studies to be included (Table 1).

**Table 1:** PICOC strategy of this study.

PICOC strategy of this study
P University students with ADHD I University services, accommodations, and intervention programmes C University students without ADHD O Benefits and improvements in university students with ADHD C University
P, Population; I, Intervention; C, Comparison; O, Outcomes; C, Context

**Figure 1:**
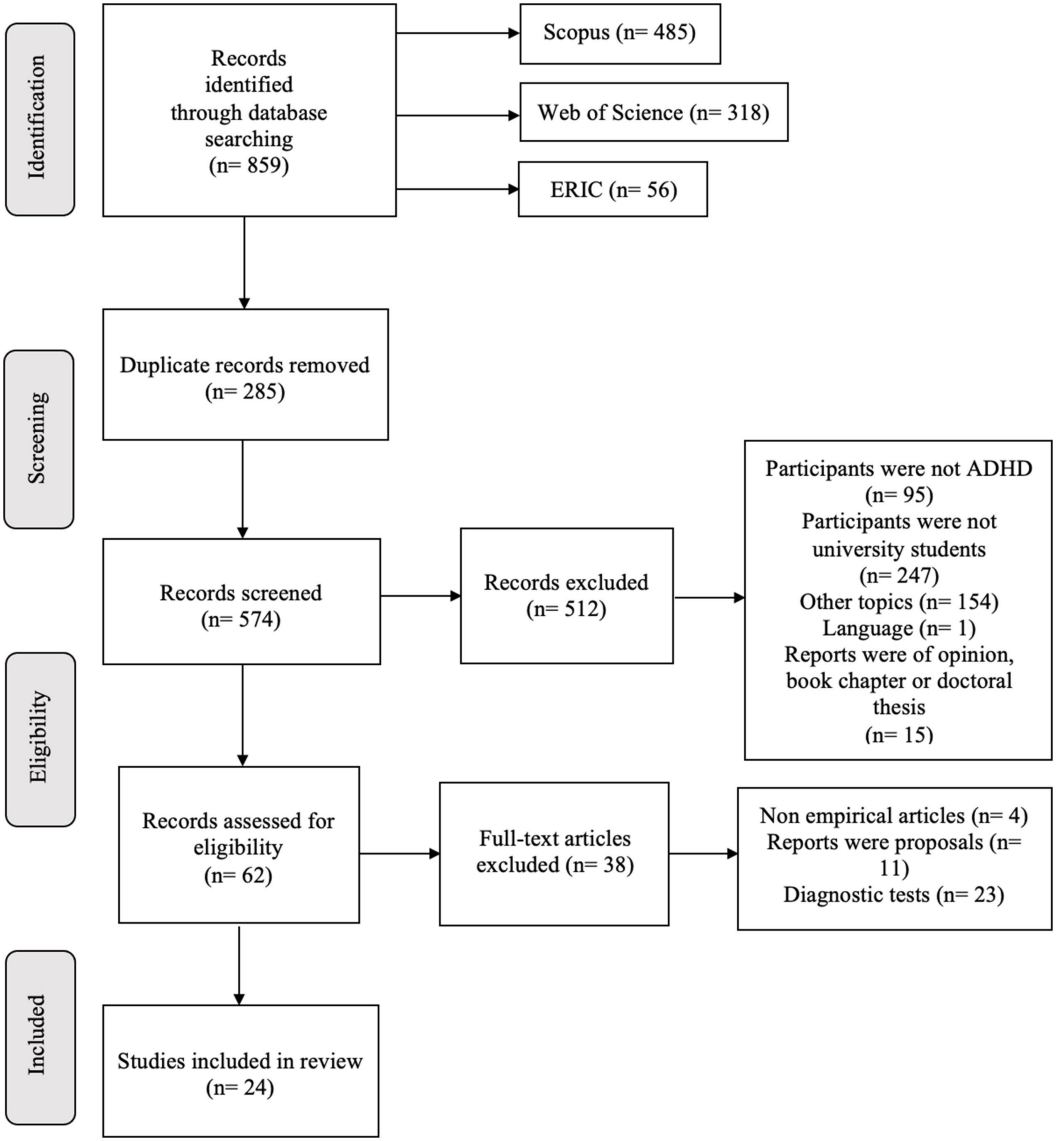
Preferred Reporting Method for Systematic Reviews (PRISMA).

This systematic review was conducted in accordance with the Preferred Reporting Method for Systematic Reviews (PRISMA) flow diagram (Moher et al., 2009) in four stages: Identification, Screening, Eligibility, and Inclusion (Figure 1). This review is based on support actions aimed at meeting the needs of university students with ADHD at an international level. Using the databases ERIC, WOS on Web of Science Core Collection, and Scopus, we analysed scientific articles from 2017 to 2022. A total of 859 articles were found using this advanced search ((ADHD OR “attention-deficit hyperactivity disorder”) AND (college OR universit* OR “higher education”) AND (support OR service* OR intervent* OR adjustment OR performance OR guidance OR accommodation OR program* OR advis*)).

The exclusion criteria used as an intermediate step in the selection where the sample did not include subjects with ADHD, the sample was not university students, the study topic did not meet our objective and, finally, the articles were non-empirical. Thus, the inclusion criteria are that the sample of the studies includes university students with ADHD that have received support, inside or outside the university, to cope with the difficulties associated with ADHD that interfere with their academic life.

A total of 859 articles were found in the different databases consulted: 485 in SCOPUS, 56 in ERIC, and 318 in WOS. Using the Mendeley bibliographic manager, with which those duplicate articles were detected (*n* = 285), the first screening of the remaining 574 articles was carried out, reading the title and the abstract, of which 512 were discarded. The second screening was carried out with the reading of the full text of the 62 articles that passed to this phase, where 38 were excluded. Finally, the studies included in this review were 24 (Figure 1).

The analysis of the data collected from the studies included in this systematic review was carried out using MAXQDA 2022 software. The code system has a total of 855 coded segments divided into 6 subcodes where the following aspects of each article are recorded: authors’ affiliation, problems and needs of university students with ADHD, objective, methodology, results, and conclusions.

## 3. Results

The final sample consisted of 24 studies. According to the publications, the sample of ADHD students belong to universities in the United States (81.8%) in 18 of the studies, with the remaining studies taking place in Belgium, United Kingdom, and Israel.

Concerning the type of actions to support university students with ADHD, two types of studies have been found. On the one hand, supporting actions offered by the universities to ADHD students (*n* = 13, 54.2%; Table 2), and, on the other hand, intervention programmes aimed at university students with ADHD outside the university settings (Table 3). Supporting actions offered by universities to ADHD students.

**Table 2:** Characteristics of the services and accommodations studies.

Authors	Participants	Method	Type of service
*DuPaul et al. (2017)	*N* = 1782 university students with ADHD and learning disabilities	Documental analysis	Tutoring Advisor Coaching
*Gormley et al. (2019)	*N* = 456 university students with and without ADHD	Survey	Academic skill assistance Advisor Tutoring
*O’Shea and Thurman (2017)	*N* = 25 employees in disability services offices	Survey	Assistance in obtaining documentation Note-taking services Transportation support Carrers services
*Gelbar and Madaus (2020)	*N* = 3,726 exams in 1517 unique courses (596 students)	Documental analysis	Test accommodations
*Jansen et al. (2017)	*N* = 214 86 students with ADHD 42 student counsellors 86 students without a disability	Survey	Test accommodations
*Kreider et al. (2019)	*N* = 52 University students with ADHD and learning disabilities	Survey	Planning and organising
*Laslo-Roth et al. (2022)	*N* = 648 529 typical students 119 students with ADHD	Survey	Distance learning Accommodations in online courses
*Mana et al. (2022)	*N* = 2,113 703 students with ADHD 370 students with learning disabilities 668 typical students	Survey	Test accommodations Social support
*Schaefer et al. (2018)	*N* = 13 Parents of university students with ADHD	Survey	Peer mentoring programme Counselling services Social support Individualized support Transportation support
*Sedgwick-Müller et al. (2022)	*N* = 50 2 university students with ADHD 48 mental health, neurodiversity, and disability practitioners, learning assessors	Survey	Academic skill assistance Individualized support
*Taylor (2018)	*N* = 335 ADHD documentation guidelines	Documental analysis	Assistance in obtaining documentation Teacher-class-assistance
*Terras et al. (2020)	*N* = 13 graduate students with disabilities	Survey	Distance learning Accommodations in online courses
*Weis and Banchemin (2020)	*N* = 1,634 university students with ADHD and learning disabilities	Intervention in university	Test accommodations

**Table 3:** Characteristics of the intervention studies.

Authors	Participants	Age *M*	Kind of intervention programme	Outcomes actions
*Anastopoulos et al. (2018)	*N* = 88 52 females 36 males	AR 17–27 *M* = 20.2	Cognitive-behavioural therapy (CBT) programme: 6–10 weeks (90-min) and received weekly individual mentoring sessions (30-min).	ADHD Executive functioning Comorbid symptoms
*Anastopoulos et al. (2021)	*N* = 250 165 females 85 males	AR 18–30 *M* = 19.7	Cognitive-behavioural therapy (CBT) programme: eight weeks (90-min) and individual mentoring sessions (30-min).	ADHD Executive functioning Adult depression Anxiety symptoms
*Bettis et al. (2017)	*N* = 62	AR 18–22 *M* = 19.67	Coping skills group: six weekly sessions. Cognitive training programme: 30 sessions (15–20 min) in six weeks.	ADHD Stress Coping skills Executive functioning Mental health symptoms
*Eddy et al. (2021)	*N* = 250 165 females 85 males	AR 18–30 *M* = 19.7	Cognitive-behavioural therapy (CBT) programme: eight weeks (90-min) and individual mentoring sessions (30-min).	ADHD symptoms and life domains Academic skills and strategies
*Gabriely et al. (2020)	*N* = 73 54 males 17 females	AR 18–40 *M* = 25.76	Mindfulness meditation: 27 participants. Eight weekly meetings (2.5-h) and a retreat day. Device-guided breathing (DGB): 35 participants. Three weeks (15-min). Control group: nine participants.	Diagnoses and interventions, age, and gender Dispositional mindfulness ADHD symptoms Breathing rate, heart rate and galvanic skin response rate
*Harris et al. (2019)	*N* = 11	AR 18–27 *M* = 22.64	Neurofeedback: eight weeks (16 sessions).	ADHD symptoms Self-concept
*Hartung et al. (2022)	*N* = 58 28 control group 30 treatment group	AR 18–32 *M* = 22.63	Organizational, Time Management, and Planning (OTMP): 10 weekly group sessions and 10 weekly individual sessions (eight sessions).	Background, developmental, and demographic data ADHD symptoms ADHD domains Organizational, time-management, and planning skills
*LaCount et al. (2018)	*N* = 27 22 intervention 15 control	-	Organizational, Time Management, and Planning (OTMP): three weeks (1-h group sessions).	ADHD domains GPA OTMP skills
*Prevatt et al. (2017)	*N* = 34	AR 18–50 *M* = 23.56	Coaching: eight weeks (50-min).	Demographic information Weekly goals Level of motivation to complete weekly objectives
*Solanto and Scheres (2020)	*N* = 38 18 ADHD 20 control	AR 19–31 *M* = 23.61 AR 18–22 *M* = 19.85	Cognitive-behavioural therapy (CBT) programme: 12 weekly sessions.	ADHD symptoms Self-concept Organization Motivation Executive functioning Time-management Anxiety Depression
*Van der Oord et al. (2020)	*N* = 58 30 intervention 28 control	AR 17–25	Cognitive-behavioural therapy (CBT) programme: 6 weeks individual (1-h).	ADHD Learning strategies Depression and problem behaviour Executive functioning

**Figure 2:**
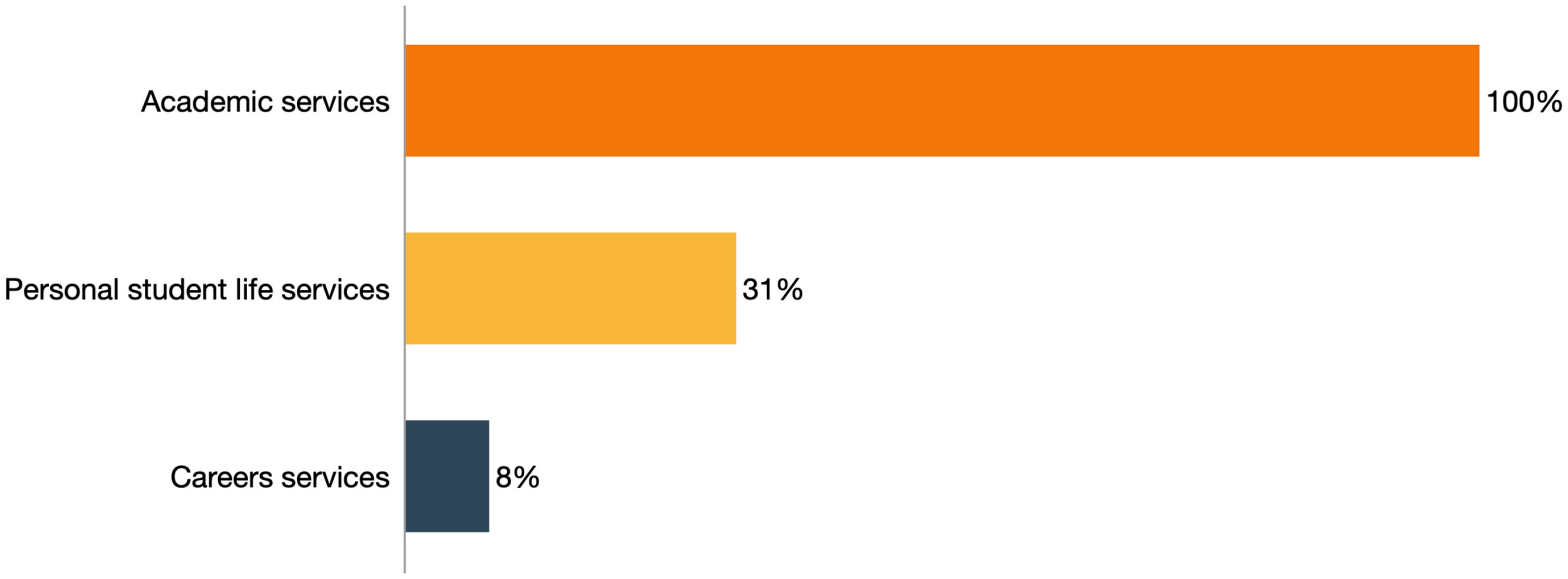
Services.

As is the case with all university students, considering the 11 studies that focus on the support provided to ADHD students by means of services and accommodations, most common are academic services, reported in all studies (100%), followed by personal student life services (31%) and less frequent, career services (8%; Figure 2).

**Figure 3:**
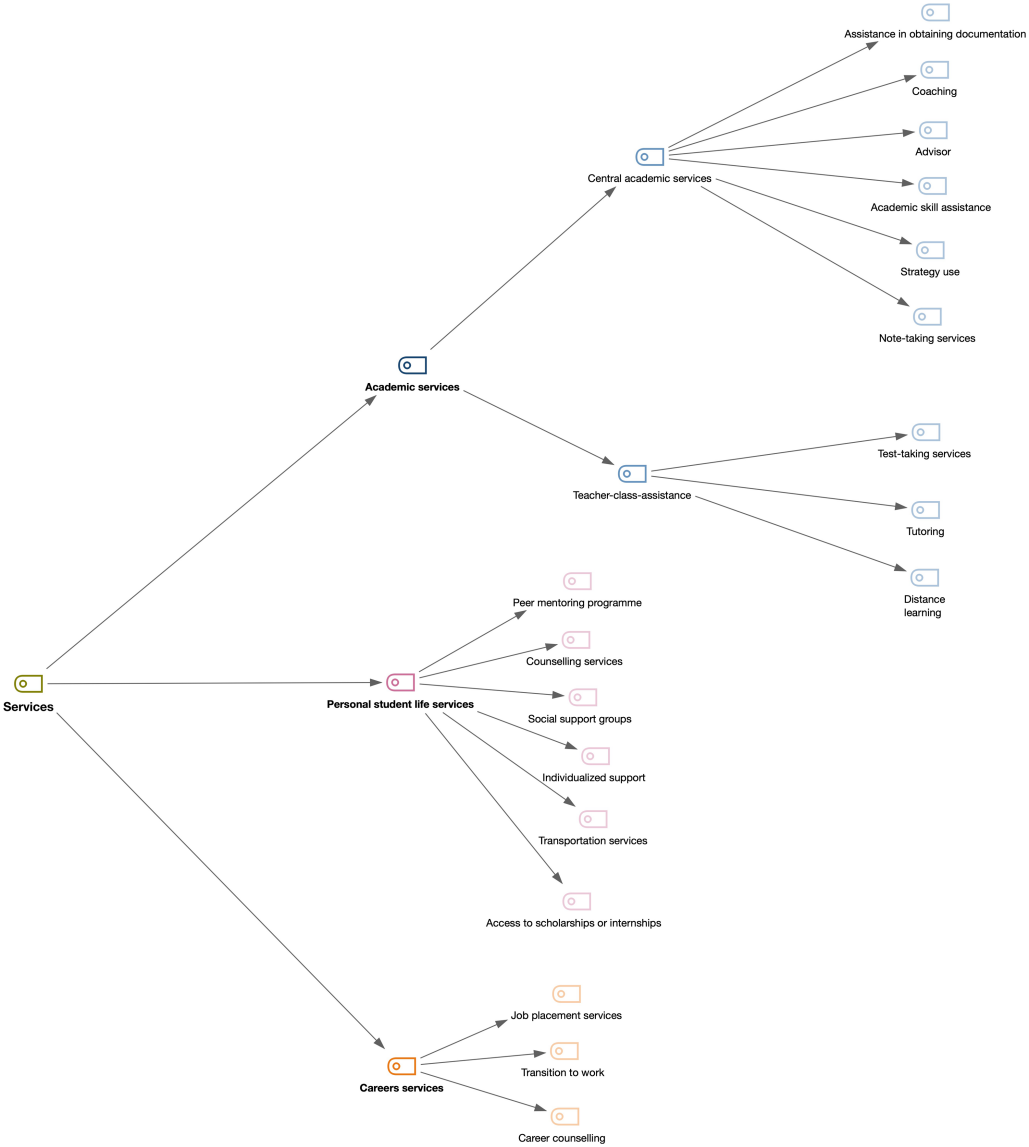
Guidance services for university ADHD students.

Thus, academic services are frequently provided by universities to students with ADHD offering accommodations, strategies, and programmes to improve their learning techniques and their exams. On the other hand, personal student life services focus on personally helping students with ADHD in the university stage with a tutor, a peer mentoring programme, individualized support, access to scholarships or internships, and the use of strategies. And, finally, career guidance services focus on the transition to work and job placement of students with ADHD (Figure 3).

Subsequently, a detailed description of the three groups is provided. Firstly, academic services can be divided into two groups according to the mode of delivery: central services and teacher-class-assistance.

### 3.1. Services and accommodations provided by universities to ADHD students

#### 3.1.1. Academic services

Figure 3 illustrates the provision of central services in the university that are intended to specifically attend to the academic needs of individuals within the institution.

#### 3.1.2. Assistance in obtaining documentation

When ADHD students have trouble arranging for an institution or organization to send documents directly to the university, several forms of assistance are available, such as (*Schaefer et al., 2018):

- Periodic automated text messages to university students informing about university events and resources (*Schaefer et al., 2018).
- Websites: more detailed webpages regarding services and documentation guidelines posted on institutional website (*Schaefer et al., 2018; *Taylor, 2018).
- Accommodation letters: is a document provided by the Office of Disability Services that explains to faculty the reasonable accommodations to be provided to a student. The letters contain course accommodations recommended by the Office of Disability Services based on the student’s disability (*O’Shea and Thurman, 2017).
- Access to scholarship or internship: services to help them apply for scholarship or internship (*Schaefer et al., 2018).

#### 3.1.3. Coaching services

These are focused on metacognitive thinking and self-determination. Students receive individual sessions with coaches with the aim of improving academic performance and self-determination, providing greater autonomy and organizational capacity as they also provide personal and emotional self-knowledge (*DuPaul et al., 2017).

#### 3.1.4. Academic advisor

An academic advisor to attend weekly academic advisory sessions with their assigned advisors offers the opportunity to reflect on their academic progress, set long-term goals and connect students with additional supports, help with transferring to a 4-year university course and information for parents of students (*DuPaul et al., 2017; *Gormley et al., 2019).

#### 3.1.5. Academic skills assistance

This academic skills assistance involves educational assistance of a professional nature to a faculty member and to students under the direction and ultimate responsibility of that faculty member (*Gormley et al., 2019; *Sedgwick-Müller et al., 2022).

#### 3.1.6. Strategy use

Planning and organising with habits and routines, reframing of challenging or frustrating experiences, activity changes, abstinence signals, creation of low stress (*Jansen et al., 2017; *Kreider et al., 2019). The use of cognitive, psychological, and socio-environmental strategies provides greater self-management in the university context in students with ADHD (*Kreider et al., 2019).

Note-taking services: Assist students in analysing and organizing information presented during the lecture and help place them on a level playing field to their non-disabled peers (*Schaefer et al., 2018).

#### 3.1.7. Teacher-class-assistance

Teacher-class-assistance is a series of academic accommodations performed by teachers to help students with ADHD in the academic field, offering adaptations in exams and tutorials so that they can adequately develop their academic skills within the university. The recommendation to perform these accommodations is often referred to university teachers by the central services (Figure 3).

#### 3.1.8. Test-taking services

These test-taking services help to select a good test location, separate rooms (decrease distractions, be able to read aloud, and reduce anxiety), provide an alternative exam format (changing the exam format from written to oral, students with ADHD do better with a format that provides word cues to jog their memory, multiple-choice or true−false questions are more AHDH-friendly as oral exams or open-book tests). Provision of extended time to complete the test, shorter sessions, breaks, and simplify language in the instructions, and provided visual time indication are the main strategies to effectively assist students in managing their time during exams (*Jansen et al., 2017; *O’Shea and Thurman, 2017; *Gelbar and Madaus, 2020; *Weis and Beauchemin, 2020; *Mana et al., 2022).

#### 3.1.9. Distance learning

University students who have experienced distance learning with this disorder reflect higher levels of loneliness as well as negative learning experiences, given the importance of therapeutic implications and university support in addition to that offered by their classmates (*Laslo-Roth et al., 2022). In the accommodations in online courses, the most used were time management and extended time, followed by autoregulation and executive functioning interventions. At all times they provided an instructor who was the link between the student and the university (*Terras et al., 2020). One of the most widely provided accommodations is extra time for students with ADHD to take their exams (*Gelbar and Madaus, 2020). A frequent measure is the adaptation in tests through separate rooms, an aspect that does not usually reduce ADHD symptoms or the anxiety that the exams produce and even reduces performance and thus grades (*Weis and Beauchemin, 2020). The importance of providing access to inclusive and individualized approaches to teaching benefits students with ADHD, not only in face-to-face classes but also in online courses (*Terras et al., 2020). Regarding accommodations, university students with ADHD suffer from academic deterioration due to their attention problems but also due to the classic teaching and evaluation methods used by professors. Therefore, they need adaptations that are individually supportive to their personal and environmental characteristics (*Jansen et al., 2017).

#### 3.1.10. Tutoring

Tutoring can be conducted either individually or in small groups, using different sessions to help students with ADHD in their organization, reading, note-taking, exam preparation, writing and improve their executive functions, and specific tutorials are provided with topics that concern students about the different subjects (*DuPaul et al., 2017; *Gormley et al., 2019). Student support services have been shown to offer academic improvement and access to tutoring is beneficial in these cases (*DuPaul et al., 2017). However, accessing these services, and specifically some adaptations, entails a series of difficulties at the time of the request due to the documentation, sometimes illegible, and especially for students with ADHD. Therefore, both the institutions and the responsible professionals should have a more inclusive language and offer a more accessible and equitable field in relation to the opportunities offered to its students (*Taylor, 2018).

In summary, academic support is the most important area for students with ADHD. Furthermore, it is essential that this support is offered directly by university teachers in the context of each subject, which implies the need for specific training for teachers. This is very important due to the fact that the transition to higher education is deficient due to poor communication between the student and the university, added to the self-management and functioning problems of university students with ADHD and the lower interaction of their parents in relation to previous academic stages (*Schaefer et al., 2018).

### 3.2. Personal student life services

Secondly, considering the personal life of students with ADHD, universities offer these students several services to help them face this stage of their lives (Figure 3).

- Peer mentoring programme is a type of tutorship with a person who has lived a specific experience, the peer mentor, and a person who is new to that experience, the peer mentee (*Schaefer et al., 2018).
- Counselling services are a collaborative process that involves the development of a confidential professional relationship that focuses on personal problems (*Schaefer et al., 2018).
- Social support services are structured meetings run by a mental health professional; for example, these groups can play an important role at a time of stress or problems (*Schaefer et al., 2018; *Mana et al., 2022).
- Individualized support is based on their needs and the barriers they face (*Schaefer et al., 2018; *Sedgwick-Müller et al., 2022).
- Transportation services (*Schaefer et al., 2018) and access to scholarships or internships help these students meet the costs of university life (*Sedgwick-Müller et al., 2022).

### 3.3. Career services

Thirdly, universities provide services that help their students transition to work, also offering help and recommendations to find these jobs and start their careers (Figure 3). Job placement services guide the students to find a post at the place they have decided on; it is a service to find a suitable job for someone, especially temporary jobs for students − for example, six-month job placements − and helps them make the transition to work, as well as offering career counselling (*Sedgwick-Müller et al., 2022). These services were used in a more limited way and less frequently in relation to those seen previously.

### 3.4. Intervention programmes to ADHD university students outside the university context

There are other studies (*n* = 11) about intervention programmes, in which the sample are university students with ADHD, but these programmes are not performed in the university setting. They include cognitive-behavioural therapy programme, organizational, time management and planning (OTMP), coaching, coping skills, device-guided slow breathing, mindfulness, cognitive training, and neurofeedback (Table 3).

**Figure 4:**
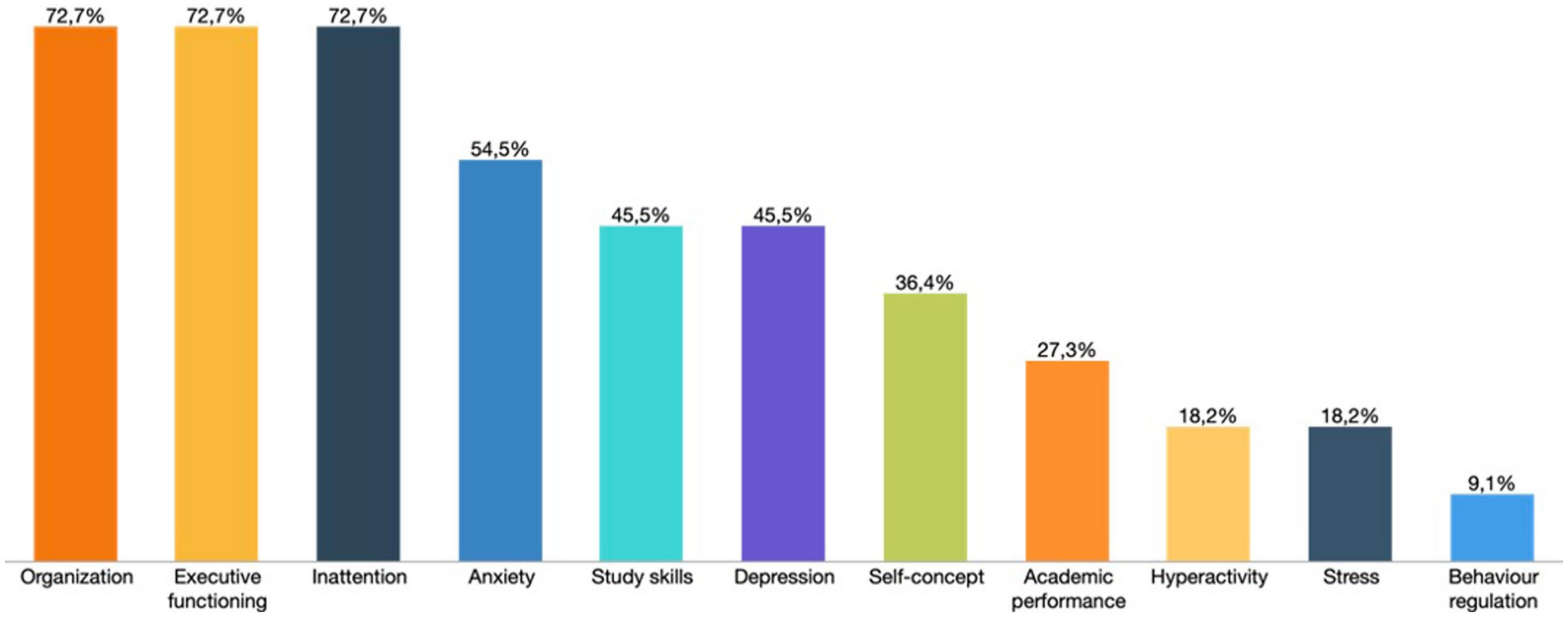
Purposes of intervention programmes.

**Figure 5:**
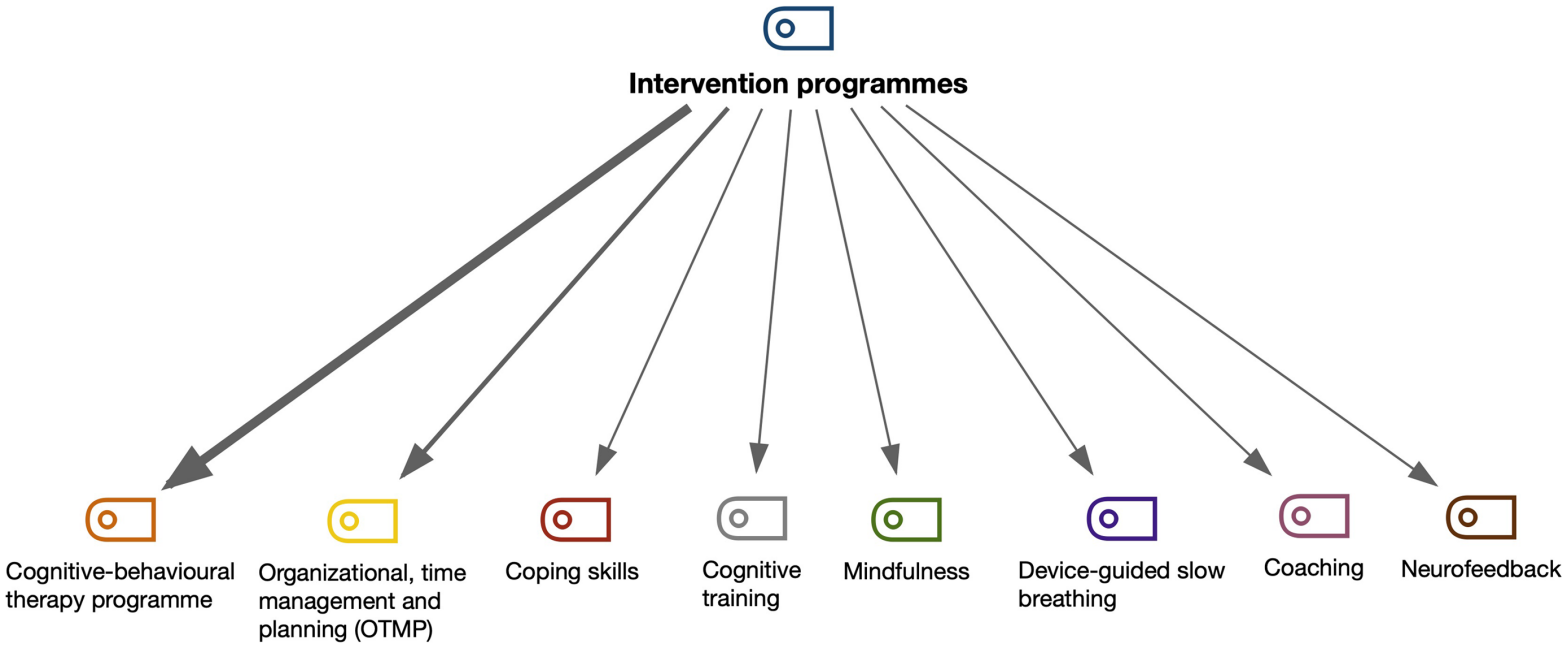
Types of intervention programmes.

These intervention programmes are differentiated according to the benefits achieved with university students with ADHD. In the different intervention programmes included in the studies analysed, the factors that obtained the greatest changes in university students with ADHD were attention, executive functioning, anxiety, and organization. These were followed by study skills, self-concept, and depression and, to a lesser extent, stress, academic performance, hyperactivity, and behaviour regulation (Figure 4). To achieve these purposes, the following intervention programmes are used (Figure 5).

#### 3.4.1. Cognitive-behavioural therapy (CBT) programme

Cognitive-behavioural therapy (CBT) programme is the most frequent programme within the studies analysed. The objectives of university students with ADHD are to increase the understanding of their own disorder through CBT sessions and tutorials, to learn about campus resources, to improve organization, planning and time management, as well as their behavioural skills to improve their executive functioning, and to teach them cognitive therapy strategies to address academic and social deterioration, the possible connections with depression and anxiety that derive from this disorder, and the emotional problems they may have. This intervention programme registered improvements in the symptoms of the disorder, especially in attention (*Anastopoulos et al., 2018; *Solanto and Scheres, 2020; *Van der Oord et al., 2020). Improvements in executive functions were also seen (*Anastopoulos et al., 2018; *Solanto and Scheres, 2020) and in some of the studies anxiety was reduced and there was less chance of depression thanks to the intervention programme (*Anastopoulos et al., 2018, 2021). It has also been shown to improve and work on study skills and strategies, making students with ADHD perform more optimal time management and improve their sense of well-being, but without influencing their academic results since they do not improve (*Eddy et al., 2021).

#### 3.4.2. Organizational, time management, and planning (OTMP)

Organizational, time management and planning (OTMP) is a new CBT intervention programme. These skills training interventions are appropriate for university students with ADHD who have organizational difficulties that may impact academic performance. These skills are based on organization, planning and time management, with the use of task lists, calendars and schedules as organizational systems, task, and motivation strategies as well as the identification of priorities. The OTMP intervention programme cause inattention to decrease but hyperactivity and impulsivity remain. The skills of these university students with ADHD improved (*Hartung et al., 2022). In another study, in addition to reducing inattention, lower levels of hyperactivity were also found, but academic grades did not show significant changes (*LaCount et al., 2018).

#### 3.4.3. Coping skills and cognitive training

The comparison of two intervention programmes for university students with ADHD.

- Coping skills whose objective was to educate students about stress to facilitate the development of adaptive coping strategies.
- Cognitive training programme is through the adaptation of the Lumosity programme in which games were implemented within the executive functions of work memory, attention-control-inhibition, or change-cognitive flexibility. Those participants with ADHD had the opportunity to understand the association of the disorder with symptoms of anxiety and depression, stressand a malfunction of executive functions.

Both intervention programmes were found to be an opportunity to reduce stress and anxiety and improve executive functioning in university students (*Bettis et al., 2017).

#### 3.4.4. Mindfulness and device-guided slow breathing

Mindfulness is defined as ‘the awareness that emerges through paying attention on purpose, in the present moment, and non-judgmentally to the unfolding of experience moment by moment’ (Kabat-Zinn, 2003, p. 143). A mindfulness intervention and guided slow breathing favours the reduction of stress in these students, this being, together with coaching, one of the most used intervention programmes to try to control ADHD symptoms. Coaching benefits those students build their own goals focusing on motivation and time management (*Gabriely et al., 2020).

#### 3.4.5. Coaching

Establishes and reinforces a set of objectives selected by the learner to set goals and foster autonomy, but also self-determination and metacognitive awareness (Parker and Boutelle, 2009). When coaching is implemented in university students with ADHD, it improves study skills and learning strategies, time management and personal goals. Through these, emotional anguish decreases and satisfaction in achieving small goals is increased (*Prevatt et al., 2017).

#### 3.4.6. Neurofeedback

Monitors automatic systems in the brain, providing feedback to the brain to increase autoregulation of brain function (Gunkelman and Johnstone, 2005). An intervention carried out through neurofeedback made the students register an improvement in their symptoms of inattention and hyperactivity as well as in their own self-concept, although there were no changes in impulsivity (*Harris et al., 2019).

## 4. Discussion

### 4.1. Summary of main findings

Throughout this review, it is confirmed that the population of university students with ADHD is present within the university community and seek help both outside and inside the university. The fact that the sample of studies that have been found include actions to help students both outside and inside the university leads us to be cautious in the discussion. Thus, on the one hand, the description of support actions being carried out by universities allows us to describe actions that have proven to be useful in the university context. Whereas, on the other hand, the intervention programs aimed at university students with ADHD, but not implemented by the universities themselves, can open a line of research to deepen on the viability of their inclusion in the university context. In relation to the origin of the sample, the most relevant countries were, in first place, the United States, followed by Belgium and Israel, presenting the different actions carried out with students with ADHD both within the universities themselves and outside of them.

On the first hand, considering the studies included in this review with actions implemented within the university context, a series of support services have been found within the classroom where the teaching staff are responsible for carrying them out. In fact, it has been shown that student support services offer academic improvement (*DuPaul et al., 2017), although it must be taken into account that on many occasions access to these services is accompanied by various difficulties at the time of application due to the documentation required, especially for students with ADHD, so both institutions should have a more inclusive language, and more equitable access in relation to the characteristics of their students, especially those with a disorder (*Taylor, 2018).

As can be seen, universities have a range of services on offer for their students with disabilities, including those with a diagnosis of ADHD. Like the general provision of services for university students, services for students with ADHD respond to the traditional areas of university counselling: academic, personal and career (Watts and Van Esbroeck, 1998). These range of services offered, in principle, satisfies the needs of ADHD students in various ways, considering not only their educational hardships, but also their familial, social, and employment circumstances (Salvi et al., 2019). However, the provision of services related to these three areas is unbalanced. Given the range of services provided by universities, the majority are in the academic sphere, universities tend to offer strategies that support the growth and development of students that favour their studies and routines. Moreover, universities also often provide services related to personal aspects of student life considering that this student’s disorder can often impact them personally and emotionally. Finally, career guidance services aimed at helping students prepare for the future workplace are also provided, though to a lesser extent. Focusing on the academic sphere, university students with ADHD suffer from academic deterioration due to their attention problems, but also due to the traditional pedagogical and assessment practices used by teachers at university. The failure to adjust these practices according to the needs of ADHD students causes considerable stress which has a significant impact on their psychological and emotional well-being (Harrison et al., 2013). Therefore, they need a series of individualised support accommodations that take into account their personal and emotional characteristics, as well as their own symptomatology due to the disorder (*Jansen et al., 2017). In this sense, the importance of facilitating access to inclusive and individualised pedagogical approaches by teachers to support students with ADHD should be considered, not only in face-to-face classes, but also with great interest in online distance learning courses (*Terras et al., 2020).

Access to tutoring is beneficial in cases of university students with ADHD (*DuPaul et al., 2017), as it is often carried out individually or in small groups with students with the same disorder, thus favouring greater attention to their needs.

One of the most frequently provided academic services to university students with ADHD is accommodations for exams, such as increased time and adapted formats to better suit their needs, as well as the use of devices to allow them to track the amount of time remaining for the exam. Also, the use of separate classrooms is offered, although it should be borne in mind that sometimes this measure does not usually reduce ADHD symptoms and test anxiety and can even contribute to marginalize them, which may lead to decreases in performance for these students, resulting in lower grades (*Weis and Beauchemin, 2020).

Regarding accommodations, university students with ADHD suffer from academic deterioration due to their attention problems but also due to the classic teaching and evaluation methods used by professors. Therefore, they need adaptations that are individually supportive to their personal and environmental characteristics (*Jansen et al., 2017). For all these reasons, it is important that university teachers are trained to meet the needs of their students with ADHD and can adapt their teaching activity, enabling the full inclusion of this group in the university environment.

On the other hand, this review included a sample of studies which t described intervention programs targeting ADHD university students, yet outside of a university setting. These interventions sought to improve symptomatic aspects of academic life and success, such as attention, executive functioning, and anxiety, as well as aiding in other skills such as organisational techniques. The acquisition process of university students with ADHD is greatly enhanced with assistance, particularly in terms of executive functioning and anxiety control (Varrasi et al., 2022).

Amongst these is coaching, which helps in self-determination and the setting of specific goals, aspects that are very helpful for students with this disorder (*Prevatt et al., 2017). Coaching has been observed as a service previously within the university context itself (*DuPaul et al., 2017), so it may be an applicable action and could overlap with the tutoring offered such as adaptations in some universities and even with another intervention programme such as mindfulness as this is considered one of the most relevant interventions for the management of this disorder.

The cognitive-behavioural therapy programme requires a specialised psychologist for its implementation, as does another of the intervention programmes analysed, neurofeedback (*Harris et al., 2019), due to its clinical aspect, requires a specialist to carry it out. This is an aspect that may be more complicated due to the lack of resources on the part of universities. The same is true for the intervention developed through coping skills and cognitive training, where a professional would be needed to deliver these sessions (*Bettis et al., 2017). Although these interventions show improvements, especially in relation to attention, hyperactivity, self-concept, executive functioning, anxiety, and depression, it would be complex to implement them within the universities itself.

In conclusion, universities carry out good practices with students with ADHD, such as services and accommodations, which are very beneficial. Some of them, such as intervention programmes, are difficult for universities to implement because they require very specific qualified staff, which would imply more economic resources for the recruitment of specialists, as well as more training for university teaching staff. Despite their economic cost, these measures would favour the inclusion of university students with ADHD.

## 5. Limitations

The limitations in conducting this systematic review were found when searching for articles, as most of them referred to students with ADHD in a school context or in relation to university students but with completely different topics, such as drug use, amongst others. Another limitation was in relation to the intervention programmes found since these are not offered by the universities themselves. In this sense, this article only provides information on the potential of these programmes in the university context, based on the resources and personnel needed. However, it must be taken into consideration that there is not yet empirical data on their applicability in a real context.

## 6. Conclusion

This paper offers a comprehensive analysis of support actions for university students with ADHD internationally. The relevance of this review confirmed that the population of university students with ADHD is present within the university, and that both the universities and external services provide a range of support measures to them. It was found that university services are concentrated in academic support, followed by personal support, and career guidance. Academic support focuses on the provision of adaptations that consists of altering the format and duration of exams. Such services are beneficial in helping students overcome the challenges posed by ADHD, but the provision is disproportionately weighted towards academic support considering their emotional challenges and potential difficulties to access the labour market. Additionally, it was also found that cognitive behavioural therapy, neurofeedback, coaching, and coping skills and cognitive training are challenging to implement within the university because they require specialist staff and resources. Nevertheless, these measures would improve inclusion amongst university students. Therefore, universities should strive to offer more appropriate and equitable access to services, as well as taking into account their need to recruit more specialist staff and resources, in order to promote the full inclusion of students with ADHD.

The findings of this study suggest potential recommendations that universities should consider in order to better support and include students with ADHD. These findings are of importance to educational practitioners, researchers and policy makers looking to enhance inclusion of these students at universities. Notably, these include academic counsellors to advise and support decision-making for these students, individualised tutoring and support groups with other students with ADHD. Consideration should be given to strategies that help these students in their organisation, planning and time management, providing active study techniques, establishing routines and realistic goals that help not only in their academic life but also socially and emotionally, thus taking care of their well-being. Participation in intervention programmes such as mindfulness or coaching are also highly recommended for this type of students in order to mediate with their symptomatology, not only through pharmacological treatments but also with a tailored intervention. In order to achieve these recommendations, strategic planning for student services at universities must be developed considering each institutional context.

However, research is needed to analyse the feasibility of including intervention programmes adapted to students with ADHD in universities. We have observed that the most used adaptation with students with ADHD is the adaptation of exams: time and format. Intervention programmes entail the need for qualified staff in universities. Providing key information on the inclusion of university students with ADHD in terms of the services, adaptations and intervention programmes offered, as well as their benefits. This systematic review provides a classification of the different actions offered to ADHD students in the university setting, which serves as a foundation to further explore applicable policies by directly engaging universities in each country. Such research can provide valuable insights into how best to support these students.

## Data availability statement

The original contributions presented in the study are included in the article/supplementary material, further inquiries can be directed to the corresponding author.

## Author contributions

M-JV, CF, and MÁ-G: conceptualization, methodology, writing review and editing, investigation, and data curation. MÁ-G: formal analysis and writing original draft preparation. M-JV and CF: supervision. All authors contributed to the article and approved the submitted version.

## Funding

This research was funded by University of León, the Spanish State Research Agency (Plan for Scientific, Technical and Innovation Research 2021-2023. Ref. PID2021-125405NB-I00), and the European Social Fund (Grant from Junta de Castilla y León, Spain, Order 21st December 2020).

## Conflict of interest

The authors declare that the research was conducted in the absence of any commercial or financial relationships that could be construed as a potential conflict of interest.

## Publisher’s note

All claims expressed in this article are solely those of the authors and do not necessarily represent those of their affiliated organizations, or those of the publisher, the editors and the reviewers. Any product that may be evaluated in this article, or claim that may be made by its manufacturer, is not guaranteed or endorsed by the publisher.
